# Dry-season transmission and determinants of *Plasmodium* infections in Jawi district, northwest Ethiopia

**DOI:** 10.1186/s12936-022-04068-y

**Published:** 2022-02-14

**Authors:** Andualem Amare, Tegegne Eshetu, Wossenseged Lemma

**Affiliations:** 1 Injibar General Hospital, Injibara, Amhara Region Ethiopia; 2 Department of Medical Parasitology, School of Biomedical and Laboratory Sciences, College of Medicine and Health Sciences, University of Gondar, P. O. Box: 196, Gondar, Ethiopia

**Keywords:** Dry-season transmission, *Plasmodium* infections, Jawi district, Ethiopia

## Abstract

**Background:**

Malaria remains a serious global public health problem, and continues to have a devastating impact on people’s health worldwide. Continuous monitoring and evaluation of current malaria transmission status in different seasons is a mainstay for the success of ongoing intervention strategies for malaria. The purpose of this study was to assess the dry-season transmission and determinants of malaria in Jawi district, northwest Ethiopia.

**Methods:**

A community-based cross-sectional study was conducted from January 13 to February 11, 2020; among selected Kebeles in the Jawi district. A multistage sampling technique was used in this study. Random and systematic sampling techniques were carried out to select *Kebeles* and each household, respectively. Light microscopy and CareStart™ Malaria HRP2/pLDH (Pf/Pv) Combo RDT were implemented to determine the prevalence of malaria. Moreover, associated risk factors in the prevalence of malaria were assessed by using a bivariate and multivariate logistic regression model.

**Results:**

A total of 219 study participants were enrolled in this study. Of the total enrolled individuals, malaria cases were found among 36 individuals with a positivity rate of 16.4% (95% CI 11.4–21.5). *Plasmodium falciparum* was the predominant species with an estimated prevalence of 87.0% in the study areas. Interrupted utilization of ITN (AOR = 4.411, 95% CI 1.401–13.880), using over 3 years older ITNs (AOR = 9.622, 95% CI 1.881–49.214), travel history (AOR = 12.703, 95% CI 2.441–66.114), living in a house with holes on the wall (AOR = 3.811, 95% CI 1.010–14.384), and living in a house with an eave (AOR = 4.23, 95% CI 1.065–16.801) significantly increased the probability of malaria positivity rate.

**Conclusion:**

Malaria is still an important public health burden among individuals in the Jawi district. Interrupted utilization of ITNs, using over 3 years older ITNs, living in a house with holes on the wall, living in a house with an eave, and travel history were identified as the risk factors of malaria. Therefore, the District health office and Health extension workers should promote daily utilization of good ITNs and improve housing conditions to reduce malaria prevalence.

## Background

Malaria remains a serious global public health problem, and continues to have a devastating impact on people’s health worldwide [1, 2]. Available data showed that despite the mortality rates of malaria were declined by 7.2% from 2015 to 2017, the incidence has been reciprocally increased by 2.2% (from 214.2 million to 219 million cases) during the same time frame. In 2019, there were an estimated 229 million malaria cases and 409,000 malaria-associated deaths occurred worldwide, and it indicates still there is an increment of malaria incidence from 2015 to 2019. The World Health Organization (WHO) Africa region is home to the bulk of the global burden of malaria with an estimated 93% malaria cases and 94% malaria-associated deaths. Of whom, children aged under 5 years accounted for 67% of malaria cases and deaths. *Plasmodium falciparum* is the predominant species with an estimated burden of 99.7% of malaria cases in the regions [3, 4].

This disproportional share of the global malaria burden in Africa is most likely due to the existence of highly efficient mosquito vector species such as *Anopheles gambiae*, *Anopheles coluzzii, Anopheles funestus,* and *Anopheles arabiensis*, which are highly specialized vectors in seeking out and feeding on human blood [5–7]. These vectors are capable to survive and are widely distributed in the region during the long dry season, where surface water crucial for their reproduction is absent for up to 7 months [8]. Vectors’ dry season diapauses and quiescence adaptive mechanisms are presumably the reason for their extended and persistent survival [9]. In addition, recent findings revealed that *Anopheles stephensi* (Asian mosquito species) has been identified in the eastern parts of Africa, and is capable of transmitting both *P. falciparum* and *P. vivax* [7, 10].

Moreover, malaria parasites capable to exist persistently in the human host for long periods allow it to bridge the dry period and it resulting the disease burden remaining high regardless of the vector abundance [11, 12]. In addition to the impact of seasonal variation on the transmission of malaria, the role of climatic changes is also crucial for vector-borne disease [13]. In this regard, recent studies confirmed that the epidemiology of malaria becoming quite complex and highly sensitive to temperature, rainfall, and topographical variation, which are the main climatic variables that directly influence vector-borne diseases’ ecosystems, including the host behavior, development, and pathogen amplification [14–20].

In Ethiopia, 68% of the country’s landmass is favourable for malaria transmission and 60% of the population lives in malarious areas. In 2017, existing evidence revealed malaria remains one of the leading causes of morbidity and mortality in the nation [21]. The transmission pattern of malaria is unstable and mainly occurs in two major (September–December) and minor (April–June) transmissions seasons [22–24]. The mortality and morbidity of malaria has been reduced in Ethiopia following the nationwide distribution of rapid diagnostic tests (RDT), artemisinin-based combination therapy (ACT), long-lasting insecticidal nets (LLINs), and indoor residual sprays (IRS) in Ethiopia since 2005. However, recent findings published in 2018 revealed that the incidence of malaria has been increasing [21, 23]. It may be due to the implementation of control measures regardless of the evidence of shifting in malaria transmission pattern and it is constrained only to previously known stable malaria transmission areas [25]. It indicates the presence of challenges ahead of the national malaria elimination strategic plan.

Moreover, the current vector control approaches mainly aim to reduce the proliferation of the vectors, reduce transmission and, therefore, reduce the number of new malaria cases through the administration of insecticides at the beginning of a rainy season [2, 26]. However, these control strategies may not be expected to achieve the successful elimination programme due to it lacking the modified diapauses and quiescence vectors’ suppression strategies to maintain their survival during the dry season period [9, 11].

Ethiopia is implementing uninterrupted and integrated public health intervention measures to successfully eradicate malaria by 2030. Undergoing an in-depth examination of malaria parasite carriage, identification, and control of both asymptomatic and symptomatic infections at a community level is crucial for the success of this long-term plan. Besides, understanding dry season malaria transmission status could be a crucial issue to inform malaria and vector control strategies to strengthen the current designed malaria elimination programmes. Since no previous published data of malaria cases in the Jawi district at a community level, this study was conducted to provide preliminary information for the scientific community. Therefore, this study intended to assess the dry season’s malaria transmission status, the major *Plasmodium* species, and identifies potential associated risk factors for its transmission in the Jawi district from a deep sample at the community level.

## Methods

### Study area and period

The study was conducted in two selected Kebeles (Wombelasi and Argabo) of Jawi district, Northwest Ethiopia, from January to February 2020. Jawi is one of the districts in the Amhara region specifically located in the Awi zone, northwest Ethiopia. According to the 2007 central statistical agency of Ethiopia report, Jawi has a total population of 79,090, of whom 9.76% are urban dwellers. Jawi district, the climate alternates with long summer rainfall (June–September) and a winter dry season (October–May) with a mean annual rainfall of 1569.4 mm. The mean temperature varies between 13.68 and 34.6 °C and the altitude ranges from 648 to 1300 m above sea level. Jawi is one of the renowned districts with high incidence and transmission of malaria as indicated by the unpublished district and zonal malaria reports.

### Study design and population

A community-based cross-sectional study was conducted to determine the dry season transmission and associated risk factors of malaria infection among dwellers in the Jawi district. All settled community members of the residents or dwellers within the selected districts were the source population of the study. In the selected districts, family members of the selected households and those who were present during the study period were included in the study. The community members who had confirmed malaria parasites from a health facility, who were taking antimalarial drugs during the data collection period, who had been treated with antimalarial drugs 1 month before participant enrollment and under 6-months-old infants were excluded.

### Sample size and sampling technique

The intended sample size was calculated using a single proportion formula. It was calculated using the nearby area’s prevalence of 16.6% [27] with a margin of error of 5% and a confidence level of 95%. Then, the calculated sample size was 213. However, 5% of the estimated sample size was added to consider the possibility of a non-response rate, and the final calculated sample size was 224. A multistage sampling technique was used to select the required sample size. Before participant enrollment, two kebeles were randomly selected using the lottery technique in the district. Then households were selected using a systematic sampling method and the study participants were chosen using a simple random sampling method.

### Data collection and laboratory methods

Prior to participant enrollment, a structured questionnaire that specifically designed to address the socio-demographic characteristics of participants and malaria risk factors was developed. The questionnaire was primarily developed in English and then translated to the local language Agewugna to check for consistency. Moreover, a pre-test was also undertaken on 5% of the total sample size among the community dwellers outside of the selected kebeles for this study, and some amendments were made. To assure and keep the consistency of data during collection, training was given for 2 consecutive days to the data collector and kebele administrators regard to the objective of the study, the data collection instrument, questionnaire administrating techniques, participant recruitment techniques among the household member, and other ethical issues by the principal investigators of this study.

### Socio-demographic characteristics and malaria risk factor assessment

A standardized, pre-tested, and structured questionnaire was administered to gather information on the marker of socio-demographic characteristics, health-related factors, environmental-related factors, IRS coverage, housing structure, and ITN availability and utilization of the community. Every head of the household (HH) in the community either female or male present at home during visiting was interviewed using the local language (Agewugna).

### Blood sample collection and laboratory procedures

Following the accomplishment of crucial data collection using the designed questionnaires, nearly 250 µL capillary blood samples from figure prick were collected aseptically from each study participant to prepare both thick and thin blood film using pre-labeled microscope slides for detection and identification of *Plasmodium* species. The prepared blood films were stained using 10% Giemsa solution for 10 min and it allows to be air-dried by putting horizontally in a slide tray at room temperature. After the stained slides were air-dried, malaria parasites were ruled out if no sexual or asexual forms of parasites are seen after reading 200 HPFs or 2500 WBCs [28].

CareStart™ Malaria HRP2/pLDH (Pf/Pv) Combo (RDT) was performed according to the manufacturer’s instructions. The kit was labeled with the respective sample code and 5 μL of blood specimen was added into the sample well of the test device. Two drops of lysis buffer were added into the buffer well to lyse the cells, release the antigen and facilitate the antigen–antibody reaction. Then, the result was read after 15–20 min and interpreted according to the manufacturer’s instruction [29].

### Data quality control

To assure the quality of data, data collectors were trained, continuously supervised and a pre-test was done to keep the validity of the questionnaire. All test procedures and interpretation of results were done based on standard operating procedures (SOPs). Expiry dates, lot №s, and handling conditions of all reagents, materials, and Malaria HRP2/pLDH (Pf/Pv) RDTs were checked daily before and during the data collection period. The quality of Giemsa staining was tested with prefixed positive and negative control blood-smeared slides from Injibara General Hospital from the beginning. Then, all the blood films were examined carefully by the trained laboratory technician and principal investigators first and re-examined blindly by senior Medical Laboratory Technologists, who are working at malaria microscopy quality assurance centre in Injibara General Hospital with updated malaria training.

### Data management and analysis

The data was coded and entered into Epi-data software to check its completeness and clearance, and then transferred to SPSS version-23 for further statistical analysis. Descriptive statistics were figured out to give a clear picture of dependent and independent variables. The association between each dependent and independent variable was explored by using binary logistic regression. Variables with a *P-value* < 0.25 in the bivariate analysis were subjected to further multivariate logistic regression analysis model to identify predictor variables and to control cofounders. Individuals diagnosed with confirmed *Plasmodium* infection using either a blood smear microscope or malaria HRP2/pLDH (Pf/Pv) RDT were considered to estimate the overall prevalence of malaria in this study (microscopy plus RDT results). Sensitivity, specificity, and predictive values for the malaria HRP2/pLDH (Pf/Pv) RDT were determined by using light microscopy as a reference. Agreement between malaria HRP2/pLDH (Pf/Pv) RDT and light microscopy in detecting both symptomatic and asymptomatic malaria were determined by Kappa value [30]. For any statistical analysis *P-*value < 0.05 was considered statistically significant.

## Results

### Socio-demographic characteristics

A total of 219 individuals were enrolled in this study. Of whom, 114 (52.1%) participants were female. The mean age of enrolled individuals was 22.3 ± 14.9 and 75% of the participants were farmers. Moreover, the remaining socio-demographic characteristics of study participants were indicated below (see Table 1).

**Table 1:** Socio-demographic characteristics of the study participants in Jawi districts; Northwest Ethiopia, January to February 2020

Variables	Total (n = 219) № (%)
Age category in years	
< 5	32 (14.6)
5–14	43 (19.6)
15–24	50 (22.8)
25–34	51 (23.3)
35–44	22 (10.1)
≥ 45	21 (9.6)
Sex	
Male	105 (47.9)
Female	114 (52.1)
Educational status of the householder	
Can’t read and write	133 (60.7)
Primary (1–8)	64 (29.2)
Secondary (9–12)	16 (7.3)
Colleges and above	6 (2.7)
Occupation of the family	
Labourer	19 (8.7)
Student	4 (1.8)
Farmer	165 (75.3)
Merchant	21 (9.6)
Civil servant	10 (4.6)

### Confirmed cases of *Plasmodium* infection

The overall prevalence of malaria infection was 16.4% ((36/219); 95% CI 11.4–21.5). Of this (32/219) 14.6% of malaria cases were identified using RDT, while 10.5% (23/219) using microscopy. Regarding *Plasmodium* species distribution, *P. falciparum* was the predominant species among confirmed cases, 28 (87.5%) by CareStartTM Pf/Pv RDT and 20 (87.0%) by light microscopy. Whereas, *P. vivax* accounted 12.5% (4 individual) by CareStartTM Pf/Pv RDT and 2 (8.7%) by microscopy, and only 1 (4.3%) participant was confirmed with *P. falciparum* and *P. vivax* mixed infection by microscopy. Statistically, significant correlation (*P* < 0.01) was observed between the prevalence of *Plasmodium* infections and the age categories. Age group 5–14 has accounted for the highest prevalence of the case with 22.7% (10/44) followed by the age group 15–24 with 21.8% (12/55) cases (see Table 2).

**Table 2:** Distribution of malaria prevalence by age group and gender in Jawi district, northwest Ethiopia, January to February 2020 (N = 219)

Variables	Microscopically confirmed cases (n, %)	Total (N)	*P*-value
Age group in years			
< 5	5 (15.6)	32	0.003
5–14	10 (23.3)	43	
15–24	11 (22.0)	50	
25–34	7 (13.7)	51	
35–44	2 (9.1)	22	
≥ 45	1 (4.8)	21	
Sex			
Male	18 (17.1)	105	0.4
Female	18 (15.8)	114	
Total	36 (16.4)	219	

### Symptomatic and asymptomatic *Plasmodium* infections

In the study area, the overall prevalence of symptomatic and asymptomatic malaria was 35.2% (25/71) and 7.4% (11/148), respectively using both RDT and microscopy. Of the total confirmed *P. vivax* individuals using RDT, 75% (3/4) were asymptomatic. But, only 37.9% (11/29) of *P. falciparum* positive study participants had no symptoms of *Plasmodium* infections. Among microscopically confirmed participants with malaria, asymptomatic cases were 50% (1/2) for *P. vivax* and 10% (2/20) for *P. falciparum* (see Table 3).

**Table 3:** Distribution of *Plasmodium* species among symptomatic and asymptomatic cases confirmed by CareStartTM Pf/Pv RDT and light microscopy, in Jawi districts; northwest Ethiopia (January to February 2020)

Malaria	RDT			Microscopy			
	*Pf* (n, %)	*Pv* (n, %)	Total	*Pf* (n, %)	*Pv* (n, %)	Mixed (n, %)	Total
Symptomatic	20 (95.2)	1 (4.8)	21	18 (94.7)	1 (5.3)	0	19
Asymptomatic	8 (72.7)	3 (27.3)	11	2 (50)	1 (25.0)	1 (25.0)	4
Overall %	28 (87.5)	4 (12.5)	32	20 (87.0)	2 (8.7)	1 (4.3)	23

### CareStart™ Pf/Pv RDT diagnostic performance using Giemsa-stained thick smear microscopy as a gold standard

Of the total 32 malaria parasite confirmed by RDT, only 19 were positive with microscopy. The remaining 13 RDT positive samples were negative by microscopy. In addition, of the total 23 microscopically detected cases, 4 malaria positives slides were negative by RDT. This made the positive predictive value of RDT low 59.4% (19/19 + 13). The negative predictive value was very high 97.9% (183/4 + 183). The overall sensitivity, specificity, positive predictive value, and negative predictive value of CareStart™ Pf/Pv RDT was 82.6%, 93.4%, 59.4%, and 97.9%, respectively. The level of agreement (Kappa value) of RDT and standard microscopy was 0.65 which is classified as a substantial agreement (*kappa* = 0.61–0.80).

### Factors associated with malaria transmission in the community

After an adjustment was made for covariates, individuals who were not daily slept under LLIN (ITN) bed nets increased the risk of malaria infection by 4.41 times more than those who were sleeping daily under the net (AOR = 4.411, 95% CI 1.401–13.880). Participants who used one LLIN bed net for more than 3 years had a 9.62 times higher chance of contacting malaria than those who used it for less than a year (AOR = 9.622, 95% CI 1.881–49.214). While individuals who used a single LLIN (ITN) for 1 to 3 years had a 6.69 times increase in malaria positivity rate (AOR = 6.691, 95% CI 1.413–31.698) when compared to those who used it for less than a year. Individuals living in a house with eave were 4.23 times more likely to be infected with malaria (AOR = 4.23, 95% CI 1.065–16.801), while holes on the wall of the house increased malaria infection by 3.811 (AOR = 3.811, 95% CI 1.010–14.384) than those individuals living in houses with no hole. Similarly, individuals who had a travel history were also significantly associated with malaria prevalence (*P* = 0.003) (see Table 4).

**Table 4:** Bivariate and multivariate analysis of factors associated with malaria prevalence in Jawi district; northwest Ethiopia, January to February 2020

List of variables	Malaria status		OR (95% CI)		*P*-value
	Neg	Pos	COR	AOR	
ITN utilization					
Yes	162	21	1		
No	15	6	3.08 (1.08–8.82)		
ITN usage					
Daily	150	16	1	1	
Usually	28	11	3.68 (1.55–8.77)	4.41 (1.40–13.88)	0.011
Duration of single ITN usage					
< 1 year	137	14	1	1	
1-3 year	26	7	2.636 (0.97–7.16)	6.69 (1.41–31.70)	0.017
> 3 years	15	6	3.914 (1.31–11.69)	9.62 (1.88–49.21)	0.007
IRS application					
Yes	175	19	1	1	
No	8	16	18.421 (6.97–8.68)		
Presence of eave					
Yes	71	22	2.479 (1.19–5.16)	4.23 (1.06–16.80)	0.04
No	112	14	1	1	
Presence of hole on the wall					
Yes	77	23	2.436 (1.16–5.12)	3.81 (1.01–14.38)	0.048
No	106	13	1	1	
Travelling to an endemic area					
Yes	9	10	7.436 (2.76–20.02)	12.70 (2.44–66.11)	0.003
No	174	26	1	1	

*AOR* adjusted odds ratio, *COR* crude odds ratio, *Neg.* negative, *Pos.* positive

## Discussion

Despite sustained control efforts have been made nearly in the past two decades to fight malaria, it remains the major cause of morbidity, mortality, and socio-economic problems in Ethiopia [31, 32]. This study revealed that malaria continues to be a major public health concern among populations who reside in the Jawi district of Awi Zone. In the present study, a considerable prevalence of malaria was found with an estimated prevalence of 16.4% % despite there is a high coverage of LLITN in the community. This result confirms that the ongoing transmission of malaria persists with considerable level during dry/low transmission season. Studies conducted in different parts of Ethiopia such as in Dangila health center in Awi zone 16.6% [27], Pawe district 14.7% [33] and in Dilla, the southern parts of Ethiopia 16% [34] showed comparable findings to the current study. On the contrary, this is inconsistent with conducted in Jiga, Jabi Tehnan district 2.8% [35], Armachiho district with 32.6% [36], and Adi Arkay 36.1% [37]. The variations could be attributed to a difference in study design, seasonality of malaria transmission, epidemiology, and climatic conditions. For example, the present study was conducted during the dry season when there is low availability of rainfall and surface water which is essential for the breeding site of mosquito vectors unlike other studies mentioned above.

The overall *Plasmodium* species proportion was found to be 87.0% *P. falciparum,* 8.7% *P. vivax*, and 4.3% mixed infections. The distribution of *Plasmodium* species in this study supported with malaria parasite distribution report in Ethiopia [38, 39], the WHO malaria reports in the Africa region [40, 41], and with other many more studies such coincide with reports in Dembia [42], Metema [43], and Ataye district, North Shoa [44]. In this study, the overall symptomatic positives were 23.4% and the asymptomatic positives were 3.4%. The finding coincides with the result investigated in North Gondar 22.3% [45] or in Hadiya Zone; Southern Ethiopia 25.8% [46], and in Jiga 2.8% [35], respectively. In this study, the prevalence of symptomatic and asymptomatic malaria infections was 35.2% and 7.4%, respectively. The prevalence of symptomatic malaria in this study was comparable with the findings in Armachiho 32% [36], in central, north, and west Gondar zones 36% [47], in low transmission areas in Amhara Region 39.4% [48]. However, this is lower than a study conducted in another country in Tanzania [49]. Besides, the asymptomatic malaria prevalence (7.4%) in this study is also comparable to the study in Tanzania [42]. On the other hand, it is found to be lower than the prevalence reported in Armachiho 68.1% [36], in Jimma Zone [50], and other countries such as Kenya and South-Eastern Bangladesh [51, 52]. In general, the variations of this study from the other studies might be due to the included study populations immune status and timing of data collection.

In this study, there was a slight increase in RDT positivity rate (7.5% or 33/438) compared with another study investigated with a much higher sample size (1.9% or 153/7878) [53]. In addition, by considering Giemsa light microscopy as a gold standard test method for malaria detection, the overall sensitivity, specificity, positive predictive value, and negative predictive value of CareStart™ Pf/Pv RDT was 83.3%, 96.9%, 60.6%, and 99.0% respectively. The sensitivity and specificity of this study are similar to the study in the Amhara region of a higher malarious area [54]. These findings were also comparable with the results of the study conducted in Cameroon [55]. In this study, the positive predictive value was lower, and this revealed that CareStart™ Pf/Pv RDT test was less specific relative to light microscopy. This might be due to the probability of false positivity results from RDT that could be caused by previously treated individuals [56] with the high eagerness of being diagnosed by lying for the exclusion criteria. A substantial agreement was found between diagnoses of the RDT and light microscopy (Kappa = 0.68) which could be consistent with the result of a study conducted in Colombia (Kappa = 0.61–0.80) [57].

In the current study, utilization of LLIN, the oldness of LLIN, house with eave, house with holes on the wall, and travel history of individuals were found to be significantly associated with malaria prevalence (*P* < 0.05). This finding is supported by different studies conducted in Ethiopia [42, 46, 58, 59]. In this study, individuals who were not daily slept under bed nets (ITNs) increased the risk of malaria infection by 4.411 times more than those who were sleeping daily under the net. This is supported by studies conducted in the Hadiya zone [46], and in Dembia district [42]. Moreover, in this study, the IRS operation coverage was 82.6% in the lowland which is again lower than the country-wide coverage [60], and it was absent or extremely lower (1.4%) in the highland areas.

In this study, the odds of malaria infection among individuals who had a history of travel were higher than those who did not. This finding is consistent with that of a study conducted in Dembia, in Northwest Ethiopia [42] and Jimma town, Southwest Ethiopia [61]. A challenge to address malaria control tools to mobile migrant workers from highland homes to organic mechanized farms in Metema–Humera lowland during the agricultural rainy season (travel history) was reported as the main reason for persistent high malaria prevalence in Central, west and North Gondar zones [62].

Above all, assessing any association for single malaria infection with a variety of risk factors in the highland area showed insignificant relation. The possible reasons for the differences of this study from the other studies could be due to differences in quality of houses, nature of the study population, the implemented malaria control and prevention strategies (LLIN and IRS coverage), and culture of ITN utilization in the study area. Even though this research was conducted at the community level considered as a strength of the study, enrolled small sample-sized study participants and unable to consider the design effect to minimize sampling errors were considered as a limitation of the study.

## Conclusion and recommendations

Malaria, especially the predominant falciparum malaria, is an important public health concern among dwellers in the study area. Individuals in the age group of 5–14 were the most vulnerable segments of the population for malaria. Despite the protective role of LLIN/ITN is confirmed against malaria, its application for a long period was found to be a risk factor. Besides, the nature of housing (presence of eave in the house and a hole in the wall of the house) and travel history were identified as risk factors for malaria. Therefore, during the implementation of malaria control and prevention activities, awareness creation should be given regarding risk factors like housing structure and utilization of single ITN for a long period alongside the distribution of ITN. Moreover, attention should be given to travellers to use the most appropriate prevention approaches. The local health office should facilitate and motivate the health extension workers to increase the utilization of ITN and create awareness about the way how and how often they should use ITNs.

## Data Availability

The data generated or analysed during this study is included in this manuscript. Other data will be available from the corresponding author upon request.

## Abbreviations

ACT: Artemisinin-based combination therapy
HRP2: Histidine rich protein 2
IRS: Indoor-Residual Spraying
ITN: Insecticide treated net
LLIN: Long-lasting insecticidal net
RDT: Rapid diagnostic test
SOP: Standard operating procedure
SPSS: Statistical Package for Social Sciences
WHO: World Health Organization
AOR: Adjusted odds ratio
COR: Crude odds ratio
